# A Standard-Based Citywide Health Information Exchange for Public Health in Response to COVID-19: Development Study

**DOI:** 10.2196/35973

**Published:** 2022-09-27

**Authors:** Bala Hota, Paul Casey, Anne F McIntyre, Jawad Khan, Shafiq Rab, Aneesh Chopra, Omar Lateef, Jennifer E Layden

**Affiliations:** 1 Tendo Systems, Inc Hinsdale, IL United States; 2 Rush University Medical Center Chicago, IL United States; 3 Centers for Disease Control and Prevention Atlanta, GA United States; 4 Wellforce Burlington, MA United States; 5 CareJourney Washington, DC United States

**Keywords:** public health, informatics, surveillance, disease surveillance, epidemiology, health data, electronic health record, data hub, acute care hospital, COVID-19, pandemic, data governance

## Abstract

**Background:**

Disease surveillance is a critical function of public health, provides essential information about the disease burden and the clinical and epidemiologic parameters of disease, and is an important element of effective and timely case and contact tracing. The COVID-19 pandemic demonstrates the essential role of disease surveillance in preserving public health. In theory, the standard data formats and exchange methods provided by electronic health record (EHR) meaningful use should enable rapid health care data exchange in the setting of disruptive health care events, such as a pandemic. In reality, access to data remains challenging and, even if available, often lacks conformity to regulated standards.

**Objective:**

We sought to use regulated interoperability standards already in production to generate awareness of regional bed capacity and enhance the capture of epidemiological risk factors and clinical variables among patients tested for SARS-CoV-2. We described the technical and operational components, governance model, and timelines required to implement the public health order that mandated electronic reporting of data from EHRs among hospitals in the Chicago jurisdiction. We also evaluated the data sources, infrastructure requirements, and the completeness of data supplied to the platform and the capacity to link these sources.

**Methods:**

Following a public health order mandating data submission by all acute care hospitals in Chicago, we developed the technical infrastructure to combine multiple data feeds from those EHR systems—a regional data hub to enhance public health surveillance. A cloud-based environment was created that received ELR, consolidated clinical data architecture, and bed capacity data feeds from sites. Data governance was planned from the project initiation to aid in consensus and principles for data use. We measured the completeness of each feed and the match rate between feeds.

**Results:**

Data from 88,906 persons from CCDA records among 14 facilities and 408,741 persons from ELR records among 88 facilities were submitted. Most (n=448,380, 90.1%) records could be matched between CCDA and ELR feeds. Data fields absent from ELR feeds included travel histories, clinical symptoms, and comorbidities. Less than 5% of CCDA data fields were empty. Merging CCDA with ELR data improved race, ethnicity, comorbidity, and hospitalization information data availability.

**Conclusions:**

We described the development of a citywide public health data hub for the surveillance of SARS-CoV-2 infection. We were able to assess the completeness of existing ELR feeds, augment those feeds with CCDA documents, establish secure transfer methods for data exchange, develop a cloud-based architecture to enable secure data storage and analytics, and produce dashboards for monitoring of capacity and the disease burden. We consider this public health and clinical data registry as an informative example of the power of common standards across EHRs and a potential template for future use of standards to improve public health surveillance.

## Introduction

Since the emergence of SARS-CoV-2, the virus that causes COVID-19, in Wuhan, China [1], a global pandemic was declared in 2020 [2], and widespread and sustained transmission was observed across the United States. As of March 23, 2022, there were 79,621,004 cases and 971,422 deaths in the United States [3].

Disease surveillance is a critical function of public health in the United States. It provides essential information about the disease burden and the clinical and epidemiologic parameters of disease and is an important element to conduct effective and timely case investigations. In addition to individual and aggregated patient data, the pandemic has required careful monitoring of health care capacity and utilization to ensure clinical care needs are met, especially in times of surges of cases that have strained capacity; ongoing surveillance of case counts can aid this need to be met

Support for the public health functions of the surveillance and epidemiology of diseases has been embedded in key national informatics initiatives in the United States for nearly 2 decades through federal programs and mandates. These efforts have included syndromic surveillance [4], electronic laboratory reporting (ELR) [5] in the meaningful use program [6] (the program in which health systems were empowered to implement electronic health records [EHRs] through multiple federal incentives), and the growth of the National Healthcare Safety Network (NHSN) [7]. These programs created linkages between hospitals, commercial laboratories, and public health across the United States that collect and organize data, often through EHR and order workflows in order to improve the timeliness and completeness of reporting.

In theory, the standard data formats and exchange methods provided by the meaningful use program should enable rapid health care data exchange in the setting of disruptive health care events, such as a pandemic. In reality, access to data remains challenging and, even if available, often lacks conformity to regulated standards [8]. The current COVID-19 pandemic revealed gaps in data liquidity (ie, data entered into a system at 1 point should be usable at other points downstream in the system) and difficulty in quickly gathering information by key stakeholders, such as policy makers and public health authorities [9].

In the early phase of the pandemic, the Chicago Department of Public Health (CDPH) and health systems in Chicago tried to address 2 major challenges: first, the ability to efficiently submit necessary clinical data elements for SARS-CoV-2–tested patients, and second, the ability to capture aggregated capacity data for resource planning in an administratively efficient manner. Despite significant EHR investments among the city’s hospitals and health systems, the inability of EHR systems to automate delivery of important data elements to public health surveillance systems meant that providers and health systems had to manually enter data into the public health reporting system. However, the high volume of patients and significant work demands on health systems limited timely and complete manual data entry. As the pandemic unfolded, multiple agencies requested bed and surge capacity information, including the NHSN, the Federal Emergency Management Agency (FEMA), the National Guard, and the Illinois Department of Public Health (IDPH), all with slightly varying data element definitions (Multimedia Appendix 1). Locally, an important aspect of capturing the resource capacity data was to monitor the surge capacity and assist with coordination of resources. The multiple reporting requirements, varying definitions, and limited mechanisms for automated, real-time submission of key resource metrics, such as bed capacities, raised concern about the ability to locally monitor the resource capacity across our systems.

In response to these challenges, the CDPH issued a public health order requiring electronic data sharing and partnered with the Rush University Medical Center to leverage existing health information technology (HIT) infrastructure for COVID-19 to develop a platform for data exchange. In this paper, we describe the technical and operational components, governance model, and timelines required to implement the public health order that mandated electronic reporting of data from EHRs among hospitals in the Chicago jurisdiction. We also evaluate the data sources, infrastructure requirements, and the completeness of data supplied to the platform and the capacity to link these sources. As an example of clinically relevant fields of interest for reporting, we compared available fields in data feeds to the *Human Infection with 2019 Novel Coronavirus Case Report* (also referred to as the COVID-19 Persons Under Investigation [PUI] Form) [10]. Finally, we reflect on success factors that enabled the rapid implementation of data sharing in the region.

## Methods

### Setting

This project was conducted by the CPDH in partnership with the Rush University Medical Center, which was made a third-party agent of the CDPH to develop and support the analytics and provide the infrastructure to support the data collection.

### Public Health Notice

On April 6, 2020, the CDPH issued public health order 2020-4 requiring hospitals in Chicago to share EHR data with the CDPH [9] for all patients tested for SARS-CoV-2. The order outlined a constrained set of data to be submitted for all SARS-CoV-2–tested patients. This order was disseminated through the CDPH’s clinical Health Alert Network (HAN), posted on the department’s website, and shared with city hospital leadership on calls. The CDPH constituted a governance committee comprising medical directors and informaticists from hospital systems in Chicago, Illinois.

### Data Feeds

ELR feeds were accessed from the Illinois National Electronic Disease Surveillance System (I-NEDSS) to provide baseline information on laboratory-confirmed cases in the city. As a result of meaningful use mandates, each positive test result for COVID-19 obtained from diagnostic laboratories and present in EHRs was being sent to I-NEDSS. These feeds contained records of patient demographics, test name, results, and dates of service and were being submitted by 88 facilities. To meet public health order 2020-4, Chicago hospitals were provided with multiple mechanisms to submit consolidated clinical data architecture (CCDA) records for SARS-CoV-2–tested patients. This included (1) a report via a secure mailbox that used the DIRECT protocol [11], a recognized data standard by the Office of the National Coordinator for Health Information Technology (ONC) for the 1-way transmission of EHRs to a centralized instance of the Epic EHR [12] for the city, or (2) a report directly to the CDPH’s instance of the Microsoft Azure cloud [13] via DIRECT or an application programming interface (API), which could receive and accept the CCDA records. In either case, the CCDAs were parsed into a database within a dedicated tenant in Azure for analytics. Additionally, a third data set of NHSN patient safety and hospital capacity was included, where hospitals were asked to either enter into a Research Electronic Data Capture (REDCap) database or send electronically to the Azure tenant. All data feeds were operational data (ie, used for purposes of public health reporting or obtained from electronic records used in patient care) and contained protected health information (PHI).

### Technical Evaluation

At the project start, we developed the requirements of a solution to collect data from sites and produced the required analytics. At the start of this project, the accepted method for COVID-19 case-related data to be submitted to health departments was the Person Under Investigation (PUI) surveillance form. These forms were available as paper forms or via survey instruments hosted on a RedCap survey tool by the IDPH. Entry was time-consuming and often incomplete due to clinical burdens. Responsibility for form completion rested with infection control practitioners or clinical staff and was considered neither timely nor complete due to competing tasks for these individuals. We evaluated the gap between the existing COVID-19 PUI form fields and the electronic data elements available in federal standard–based data feeds and developed a crosswalk of reporting requirements to ensure that the data set could function as a reporting gateway for sites and reduce the burden of reporting. Feeds evaluated were ELR, CCDA, and Fast Healthcare Interoperability Resource (FHIR, pronounced as *fire*) fields. Missingness and usefulness were evaluated among CCDA and ELR feeds. Missingness refers to whether data are present in the field. Usefulness refers to clean and complete information in the data field. Data were labeled not useful if any of the following were present in their respective fields: “unknown” in race, ethnicity, or other string fields; the presence of PO boxes, unknown, homeless, or not applicable (N/A) for an address; the absence of a telephone number, an implausible number (eg, 111-1111 or 999-999-9999), or not enough numbers for the phone number; and less than 5 digits or 99999, 00000, or text (eg, “UUUUU”) for zip codes. Records were deduplicated using name and date of birth. The record match rate between CCDA and ELR data feeds was assessed: a deterministic match process using an exact match of characters in 12 different combinations (“keys”) of last name, first name, and date of birth was implemented, which has been shown to have efficacy in matching using surveillance registries [14]. We did not attempt to resolve close matches. For the 3 fields demonstrating the most missing or low-quality data (ie, race, ethnicity, and telephone number), we examined the additional completeness to ELR feeds by augmenting with CCDA data; this was accomplished by using complete and useful data when ELR feeds were missing for an individual person.

### Ethics

This investigation was part of the ongoing public health response to COVID-19. This activity was reviewed by the Centers for Disease Control and Prevention (CDC) and was conducted consistent with applicable federal law and CDC policy (see, eg, 45 C.F.R. part 46.102(l)(2); 21 C.F.R. part 56; 42 U.S.C. §241(d); 5 U.S.C. §552a; 44 U.S.C. §3501 et seq.).

## Results

### State Surveillance System Baseline Reporting

In Chicago, a significant proportion of reported cases of SARS-CoV-2 infections are reported through ELR. As of June 30, 2020, ELR alone provided 73.7% of cases, while ELR combined with other modalities (eg, submission of a case report from a hospital or health care provider to I-NEDSS) accounted for 94% of reported cases. ELR data reported key fields requested in the COVID-19 PUI form (Table 1) but not all; data fields routinely absent from ELR feeds included travel histories, clinical symptoms, and comorbidities.

**Table 1:** Crosswalk table to compare coverage of Human Infection with 2019 Novel Coronavirus Case Report form fields and ELR^a^, the CCDA^b^, and FHIR^c^.

CDC^d^ PUI^e^ form field		Covered in CCDA	Covered in ELR	Covered in FHIR	Covered in other data sources
**What is the current status of this person?**					
	PUI: testing pending^f^	Yes (lab test and result information)	N/A^g^	Yes	N/A
	PUI: tested negative^f^	Yes (lab test and result information)	N/A	Yes	N/A
	Presumptive case (positive local test): confirmatory testing pending	N/A	N/A	N/A	N/A
	Presumptive case (positive local test): confirmatory tested negative	N/A	N/A	N/A	N/A
	Laboratory-confirmed case	Yes	Yes	Yes	N/A
	Report date of PUI to CDC	N/A	N/A	N/A	N/A
	Report date of case to CDC	N/A	N/A	N/A	N/A
	County of residence	Yes	Yes	Yes	N/A
	State of residence	Yes	Yes	Yes	N/A
	Ethnicity	Yes	Yes	Yes	N/A
	Race	Yes	Yes	Yes	N/A
	Sex	Yes	Yes	Yes	N/A
	Date of birth	Yes	Yes	Yes	N/A
	Age	Yes	Yes	Yes	N/A
Was the patient hospitalized? Date?		Yes	N/A	Yes	ADT^h^ or Census data
Was the patient admitted to the ICU^i^?		N/A	N/A	Yes	ADT or Census data
Did the patient receive mechanical ventilation (MV) or intubation? Days of MV?		N/A	N/A	N/A	Custom report
Did the patient receive extracorporeal membrane oxygenation (ECMO)?		N/A	N/A	N/A	Custom report
Did the patient die as a result of this illness? Date?		Yes	N/A	Yes	ADT
Date of first positive specimen collection		Yes	Yes	Yes	N/A
Did the patient develop pneumonia?		Yes	N/A	Yes	N/A
Did the patient have acute respiratory distress syndrome?		Yes	N/A	Yes	N/A
Did the patient have another diagnosis/etiology for their illness?		N/A	N/A	N/A	N/A
Did the patient have an abnormal chest X-ray?		N/A	N/A	Yes^f^	N/A
Symptoms present during course of illness: (symptomatic/asymptomatic/unknown)		N/A	N/A	N/A	N/A
Symptom onset date		N/A	N/A	N/A	N/A
Symptom resolution date		N/A	N/A	N/A	N/A
Is the patient a health care worker in the United States?		N/A	N/A	N/A	N/A
Does the patient have a history of being in a health care facility (as a patient worker or visitor) in China?		N/A	N/A	N/A	N/A
**In the 14 days prior to illness onset, did the patient have any of the following exposures (check all that apply)?**					
	Travel to Wuhan	N/A	N/A	N/A	N/A
	Travel to Hubei	N/A	N/A	N/A	N/A
	Travel to mainland China/other non-US country	N/A	N/A	N/A	N/A
	Community contact with another lab-confirmed COVID-19 case	N/A	N/A	N/A	N/A
	Any health care contact with another lab-confirmed COVID-19 case (patient/visitor/health care worker [HCW])	N/A	N/A	N/A	N/A
	Exposure to a cluster of patients with severe acute lower respiratory distress of unknown etiology	N/A	N/A	N/A	N/A
	Household contact with another lab- confirmed COVID-19 case	N/A	N/A	N/A	N/A
	Animal exposure	N/A	N/A	N/A	N/A
	If the patient had contact with another COVID-19 case, was this person a US case?	N/A	N/A	N/A	N/A
**Under what process was the PUI or case first identified (check all that apply)?**					
	Clinical evaluation leading to PUI determination	N/A	N/A	Yes^f^	N/A
	Contact tracing of the patient	N/A	N/A	N/A	N/A
	Routine surveillance	N/A	N/A	N/A	N/A
	Epidemic Information Exchange (EpiX) notification of travelers, if checked	N/A	N/A	N/A	N/A
	Unknown	N/A	N/A	N/A	N/A
	Other (specify)	N/A	N/A	N/A	N/A
**Symptoms**					
	Fever >100.4°F (38°C)	N/A	N/A	Yes^f^	N/A
	Subjective fever (felt feverish)	N/A	N/A	Yes^f^	N/A
	Chills	N/A	N/A	Yes^f^	N/A
	Muscle aches (myalgia)	N/A	N/A	Yes^f^	N/A
	Runny nose (rhinorrhea)	N/A	N/A	Yes^f^	N/A
	Sore throat	N/A	N/A	Yes^f^	N/A
	Cough (new onset or worsening of chronic cough)	N/A	N/A	Yes^f^	N/A
	Shortness of breath (dyspnea)	N/A	N/A	Yes^f^	N/A
	Nausea or vomiting	N/A	N/A	Yes^f^	N/A
	Headache	N/A	N/A	Yes^f^	N/A
	Abdominal pain	N/A	N/A	Yes^f^	N/A
	Diarrhea (≥3 loose/looser-than-normal stools/24-hour period)	N/A	N/A	Yes^f^	N/A
	Other	N/A	N/A		N/A
**Pre-existing medical conditions**					
	Chronic lung disease (asthma/emphysema/chronic obstructive pulmonary disease [COPD])	Yes	N/A	Yes	N/A
	Diabetes mellitus	Yes	N/A	Yes	N/A
	Cardiovascular disease	Yes	N/A	Yes	N/A
	Chronic renal disease	Yes	N/A	Yes	N/A
	Chronic liver disease	Yes	N/A	Yes	N/A
	Immunocompromised condition	Yes	N/A	Yes	N/A
	Neurologic/neurodevelopmental intellectual disability	Yes	N/A	Yes	N/A
	Other chronic diseases	Yes	N/A	Yes	N/A
	If female, currently pregnant	N/A	N/A	N/A	N/A
	Current smoker	Yes	N/A	Yes	N/A
	Former smoker	Yes	N/A	Yes	N/A
	Respiratory diagnostic testing test (respiratory virus testing panel information)	Yes	N/A	Yes	N/A
**Specimens for COVID-19 testing**					
	Nasopharyngeal swab/oropharyngeal swab/sputum/other (specify)	N/A	N/A	N/A	N/A

^a^ELR: electronic laboratory reporting.

^b^CCDA: consolidated clinical document architecture.

^c^FHIR: Fast Healthcare Interoperability Resources.

^d^CDC: Centers for Disease Control and Prevention.

^e^PUI: Person Under Investigation.

^f^If notes are shared through FHIR.

^g^N/A: not applicable.

^h^ADT: admission, discharge, and transfer.

^i^ICU: intensive care unit.

### Response to the Public Health Notice

On April 6, 2020, Public Health Order 2020-4 was shared via the HAN in Chicago with all eligible institutions (ie, health systems within the Chicago City borders). The order mandated the sharing with the CDPH of 3 main data types: (1) ELR feeds of SARS-CoV-2–tested individuals, which were an existing state mandate; (2) CCDA records from hospitals for SARS-CoV-2–tested patients; and (3) NHSN capacity module reporting, which was asked to be sent centrally to the CDPH. These data were requested to be sent at a minimum once per day by 10:00 a.m. US Central Time. Sites also provided contact information for key Rush University Medical Center personnel who were leading the implementation. A series of calls with hospital technical staff were conducted by the Rush University Medical Center chief information officer to introduce the project, review the rationale, and describe technical approaches.

An Azure-hosted and isolated environment was established, with 5 individual modalities for connectivity, all feeding into a centralized data hub from more than 40 organizations and hundreds of thousands of transactions per week. Over the next 30 days, all sites were approached to initiate data sharing; a CDPH data governance committee comprising chief medical officers and chief medical informatics officers from select institutions was created through which issues could be discussed and additional roadmaps could be generated; collaboration with Epic and Cerner EHR developers was established and mechanisms for enterprise scale sharing created; and data were sent centrally to the CDPH Azure instance.

### Technical Architecture

**Figure 1:**
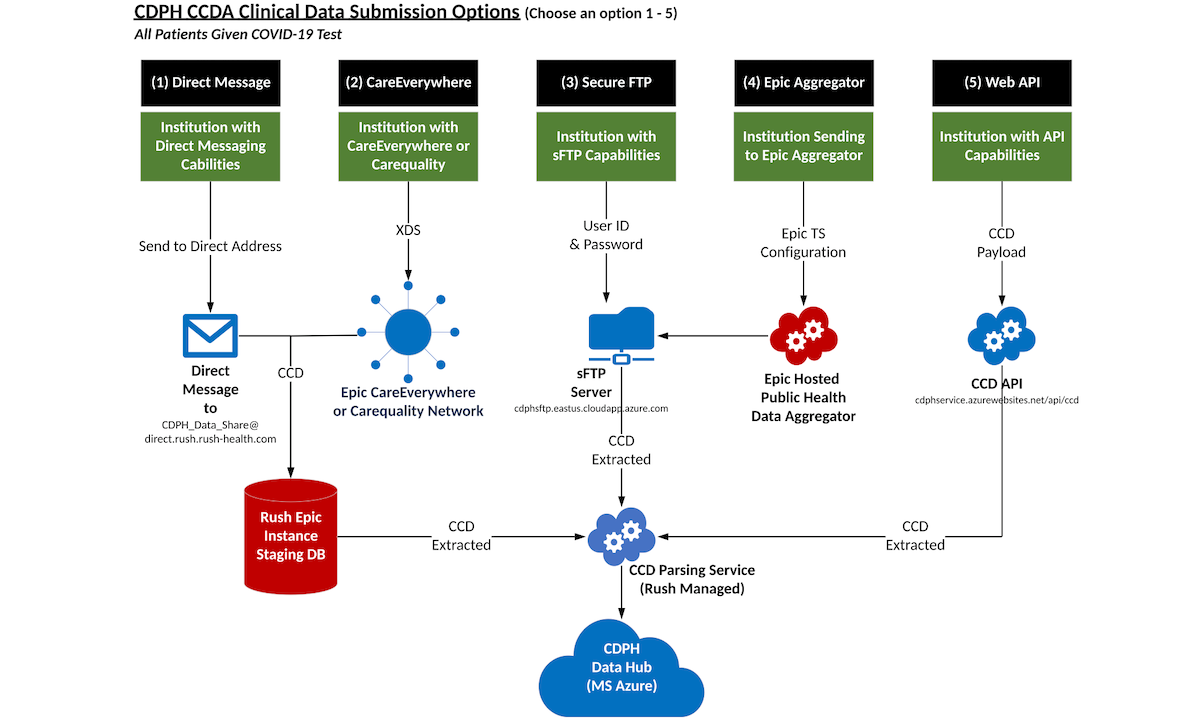
High-level architecture of the CDPH data hub CCDA submission options. API: application programming interface; CCD: continuity of care document; CCDA: consolidated clinical data architecture; CDPH: Chicago Department of Public Health; sFTP: secure file transfer protocol; TS: technical services; XDS: cross-enterprise document sharing.

An overview of the technical architecture of the project is shown in Figure 1 and was designed to maximize security and privacy of data, keeping the CDPH at the center of data use. At a high level, because of the tools from meaningful use adoption, connections existed between stakeholders in the system, which could support secure file sharing with the ability to choose records based on criteria. These tools included (1) standard-based representation of clinical data (eg, CCDA), (2) secure methods of data transport both within and external to EHR systems (eg, CareEverywhere within Epic, DIRECT mailboxes, and API-based authenticated pathways), and (3) existing implementation of complex public health rules within EHRs to identify cases and submit to public health (eg, ELR). Limited mapping of semantic content was required because data shared between health systems and public health used CCDA and Health Level Seven International (HL7) meaningful use standards, with content mapped to standard vocabularies before submission. Vocabularies used were HL7 race, gender, and ethnicity categories; *International Classification of Diseases, Tenth Revision* (ICD-10) and Current Procedural Terminology (CPT) codes for diagnoses and procedures; and Logical Observation Identifiers Names and Codes (LOINC) for lab test names. The cloud-based environment was Health Insurance Portability and Accountability Act (HIPAA) certified, and data were encrypted at rest and in transit. DIRECT mailboxes leveraged certificate-based encryption, and API pathways used hypertext transfer protocol secure (https).

A cloud-based environment was created that was totally isolated from the Rush University Medical Center EHR instance and patient records. This environment was built to support over 40 organizations within the city of Chicago and designed to scale across public health departments.

ELR data feeds were the most straightforward to use in the model, as existing connections between hospital systems were present for communicable disease reporting. Hospitals were required to implement new logic at the outset of SARS-CoV-2 infections in Chicago to identify and report lab-identified cases of COVID-19 to the CDPH and tested patients as those are PUIs. ELR feeds are submitted to the state public health agency, which makes these available to the CDPH and local health departments.

To isolate data, the Rush University Medical Center created an isolated Azure Data Repository, including Microsoft Azure SQL Warehouse, and a CosmosDB for survey forms data was created. We found that not all cross-enterprise document sharing (XDS) and DIRECT messages could avoid our EHR instance, so we needed to identify a way to enforce separation of data. We addressed this by pulling data from the Epic staging area. In addition, infrastructure components were created that included an XDS service server, DIRECT message communication, a continuity of care document (CCD) to the FHIR service, and integration with Epic via a community health aggregator. Google Apigee handled the API layer, and services were handled behind Apigee for token control. Data collection via manual entries was handled via REDCap forms with integration via the API into the Azure environment.

### Governance

Data governance was planned from the project beginning to aid in consensus and principles for data use. Although the local health department, with its public health orders, was a necessary recipient and data user, participants recognized the value of a larger sharing initiative, plus site participation to engage on use cases and mechanisms to leverage the information. The governance committee comprised the chief medical officer (CMO), the chief medical informatics officer (CMIO), or the technical lead from each of the 12 sites. These leaders also brought content and guidance back to site participants and sought to bridge varying degrees of internal technical capabilities among systems. The committee met weekly and helped to build trust among participating sites. General principles were modeled after rules implemented for use of Centers for Medicare & Medicaid Services (CMS) data [15] and were established among sites through this committee. These principles were:

- Openness: promoting and facilitating the open sharing of knowledge about COVID-19 data
- Communication: promoting partnerships across the region to eliminate duplication of effort, a source of truth for regional data that may enable reducing administrative burden, and a valuable regional and national resource
- Accountability: ensuring compliance with approved data management principles and policies and understanding the objectives of current and future strategic or programmatic initiatives and how they impact, or are impacted by, existing data management principles and policies and current privacy and security protocols

### Reporting of Bed, Supply, and Clinical Capacity

Metrics mandated for reporting to multiple agencies and groups for Chicago hospitals at the time of the hub creation are shown in Multimedia Appendix 1. In this inventory, over 100 measures to 4 systems were required: the NHSN, EMResource, FEMA, and the Illinois National Guard. The systems measure bed usage, emergency department (ED) usage, ventilator usage, supply usage and need, and laboratory testing. Of note, 57 different bed usage measures alone exist among the 4 systems. Although metrics shown had similar definitions, these still require separate administrative efforts for the data collection and reporting.As of July 31, 2020, 14 hospitals in Chicago were reporting data to the hub. For bed capacity reporting, 7 were reporting NHSN data through manual data submission, 2 were reporting through electronic queries from their EHRs with electronic submission to the hub, and 14 were submitting to EMResource.

### Completeness of Reporting via ELR and the CCDA

Data from 86,499 persons from CCDA records among 14 facilities and 408,741 persons from ELR records among 88 facilities were submitted, representing records meeting criteria to be reported under the public health order. Table 2 shows the volume and completeness of data feeds related to COVID-19, as obtained from CCDA and ELR feeds. Patients with records in these feeds were those diagnosed through reverse transcription–polymerase chain reaction (RT-PCR) testing with a Chicago address through July 31, 2020. For those individuals with more than 1 test reported, data were deduplicated. Among individuals with CCDA records submitted, 11,491 (13.3%) had a positive test compared to 53,968 (13.2%) among ELR feeds.

We examined CCDA and ELR data fields for completeness defined as a populated (ie, nonmissing) data field and usefulness defined as clean, complete information in a data field. CCDA data provided an improvement in the quality of data available for surveillance. ELR feeds had gaps in the usability or quality of race and ethnicity data (race: n=382,097, 93.5%, nonmissing and n=215,273, 52.7%, useful; ethnicity: n=333,122, 81.5%, nonmissing and n=165,715, 49.7%, useful). The CCDA was highly complete with <5% missing information in data fields for all records types except for patient phone numbers. In addition, 99.2% of CCDA data was nonmissing for both race (n=85,794) and ethnicity (n=85,799), and 82.5% of CCDA data was useful for race (n=71,345) and 79.2% for ethnicity (n=68,507). The CCDA, although covering fewer records, also had information related to encounters and hospitalization, and the presence of comorbidities.

CCDA and ELR data feeds were matched by name and date of birth among 90.6% (n=78,378) of patients in the CCDA field. With matching, some improvement in data completeness for the 3 most incomplete fields was noted for ELR data: race, ethnicity, and telephone number. Of the 78,378 matched CCDA and ELR feeds, ELR race data alone improved from 79.4% to 88.5% (n=62,232-69,365) useful data with the CCDA, while ELR ethnicity data alone improved from 58.2% to 86.7% (n=45,616-67,954) with the CCDA. Telephone number data were 78.6% (n=321,121) complete in ELR; combining the CCDA and ELR improved completeness to 80.0% (n=326,993). In addition, for the matched set, complete hospitalization and comorbidity information was present.

**Figure 2:**
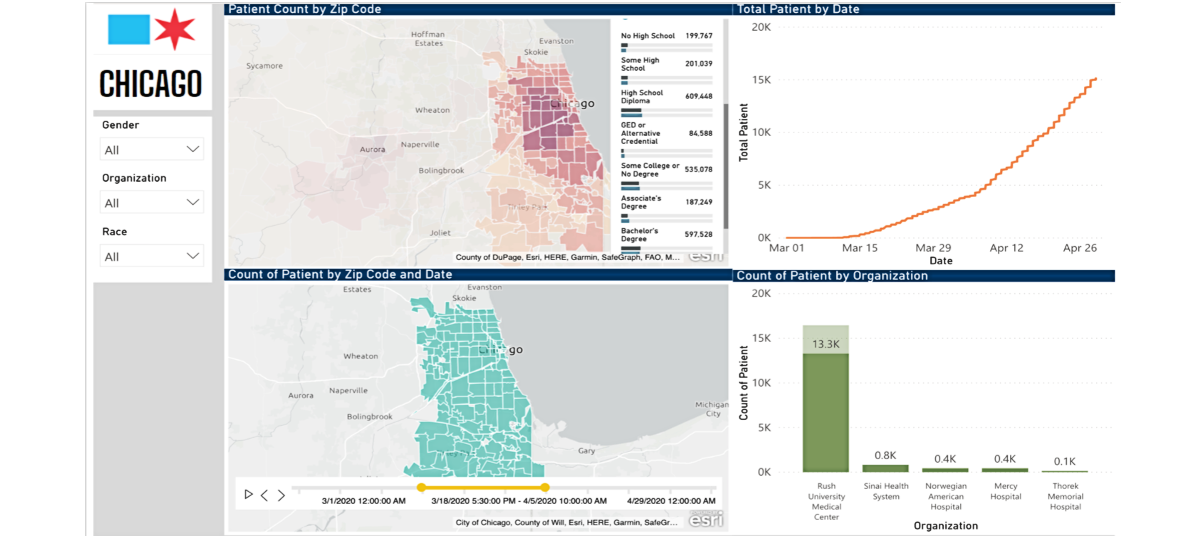
Epidemiologic dashboards for assessment of outbreak, CDPH data hub. CDPH: Chicago Department of Public Health.

For presentation, data were displayed on a dashboard available for CDPH analysts, via the Microsoft Azure Power BI platform, and are shown in Figure 2. Data from the dashboard were shared to contributing hospitals over a business intelligence portal hosted by the Rush University Medical Center and via email of bed capacity reports and analytic descriptions of case counts by subgroup. Bed capacity reports aligned with bed types listed in Multimedia Appendix 1: critical care versus general medical, and overall capacity versus COVID-19 utilization.

**Table 2:** Completeness of data submitted via the CCDA^a^ and ELR^b^.

Data field		CCDA data (N=86,499), n (%)	ELR data (N=408,741), n (%)
Lab-confirmed SARS-CoV-2		11,491 (13.3)	53,968 (13.2)
**Facility name/reporting lab**			
	Nonmissing	86,499 (100.0)	408,737 (100.0)
	Useful	86,499 (100.0)	408,463 (99.9)
**Patient first name**			
	Nonmissing	86,499 (100.0)	408,732 (100.0)
	Useful	86,499 (100.0)	408,717 (100.0)
**Patient last name**			
	Nonmissing	86,499 (100.0)	408,732 (100.0)
	Useful	86,497 (100.0)	408,718 (100.0)
**Patient date of birth**			
	Nonmissing	86,489 (100.0)	408,270 (99.9)
	Useful	86,480 (100.0)	407,730 (99.9)
**Patient sex (male/female/unknown)**			
	Nonmissing	86,416 (99.9)	408,540 (100.0)
	Useful	86,405 (99.9)	398,590 (97.5)
**Patient race**			
	Nonmissing	85,794 (99.2)	382,097 (93.5)
	Useful	71,345 (82.5)	215,273 (52.7)
**Patient ethnicity**			
	Nonmissing	85,799 (99.2)	333,122 (81.5)
	Useful	68,507 (79.2)	165,715 (49.7)
**Patient address**			
	Nonmissing	86,498 (100.0)	385,073 (94.2)
	Useful	85,471 (98.8)	384,000 (93.9)
**Patient city**			
	Nonmissing	86,499 (100.0)	408,741 (100.0)
	Useful	86,499 (100.0)	408,741 (100.0)
**Patient zip code**			
	Nonmissing	86,377 (99.9)	408,026 (99.8)
	Useful	86,375 (99.9)	407,918 (99.8)
**Patient home or cell phone^c^**			
	Nonmissing	20,712 (23.9)	321,121 (78.6)
	Useful	20,712 (23.9)	319,974 (78.3)
**Test name (open text field)**			
	Nonmissing	86,499 (100.0)	408,694 (100.0)
	Useful	86,499 (100.0)	408,694 (100.0)
**Logical Observation Identifiers Names and Codes (LOINC)**			
	Nonmissing	0	408,741 (100.0)
	Useful	0	408,727 (100.0)
**Test results (raw feed/open text field)**			
	Nonmissing	55,783 (64.5)	405,650 (99.2)
	Useful	55,235 (62.1)	396,110 (96.9)
**Test results (interpreted from open text field^c^)**			
	Nonmissing	86,499 (100.0)	408,741 (100.0)
	Useful	86,499 (100.0)	408,741 (100.0)
**Test date**			
	Nonmissing	83,999 (97.1)	408,046 (99.8)
	Useful	83,999 (97.1)	408,046 (99.8)
**Hospitalization (yes/no)^d^**			
	Nonmissing	86,499 (100.0)	0
	Useful	86,499 (100.0)	0
**Comorbidities**			
	Nonmissing	86,499 (100.0)	0
	Useful	86,499 (100.0)	0

^a^CCDA: consolidated clinical document architecture.

^b^ELR: electronic laboratory reporting.

^c^CCDA completeness represents at least 1 phone number from either the home or cell data fields; the ELR feed has 1 phone field, so home and fields cell are not differentiated. “Nonmissing” refers to a populated data field. “Useful” refers to clean, complete information in a data field. Data were labeled not useful if any of the following were present in their respective fields: “unknown” in race, ethnicity, address, or other string fields; for address, the presence of PO boxes, unknown, homeless, or N/A; for phone, an implausible number (eg, 111-1111 or 999-999-9999), or less than 10 numbers; and for zip code, less than 5 digits or 99999, 00000, or letters (eg, “UUUUU”).

## Discussion

### Principal Findings

In this report, we described the development of a citywide public health data hub for the surveillance of SARS-CoV-2 infection in Chicago, Illinois. We were able to assess the completeness of existing ELR feeds, augment these feeds with CCDA documents, establish secure transfer methods for data exchange, develop a cloud-based architecture to enable secure data storage and analytics, and produce meaningful dashboards for the monitoring of capacity and disease burden.

An underlying need in public health that drove our work was an aim to improve the automation, completeness, and usefulness of data submitted to public health agencies. The work builds on the known utility of ELR with improved data quality. ELR, or the submission electronically of laboratory tests to a public health department through implementation of business logic for detection, has been found in multiple studies to improve the timeliness and completeness of reporting [16-20] at potentially lower costs [21]. A review prior to widespread electronic reporting use found that despite legal mandates for reporting, passive surveillance yielded completeness rates of 23%-81% for communicable diseases with higher rates for active surveillance [22] and timeliness of reporting between 10 and 13 days after laboratory result dates [23]. ELR systems have improved the reporting of data to public health for surveillance, with the volume and timeliness of reporting improving 2.3-4.4-fold and 3.8-7.9 days earlier, respectively [24]. ELR has been a major advance in that it can improve the completeness of reporting over what is found through passive surveillance [21,25].

ELR data have been hampered by ongoing issues with completeness. In prior reports, ELR data have been found to vary in their completeness: the completeness of fields reported via ELR within basic HL7 v2.x messages ranges from 38% (race) to 98% (date of birth) [25]. To increase completeness, improvements have been proposed: (1) increase in mandatory fields in ELR HL7 v2.x messages [24]; (2) augmenting of ELR feeds with data from a health information exchange, which improves completeness for race to 60% [25]; and (3) electronic case report forms that are completed through either automated data capture or manual completion [26]. Significant limitations in case reporting have been identified during the COVID-19 pandemic, including limited data on key variables such as age, race/ethnicity, hospitalization, and intensive care unit (ICU) status [27].

We also found that ELR data do not provide all the information needed for adequate case investigation. Demographic and risk factor information may not be complete in the HL7 feeds for ELR, and case report forms continue to play a critical role in the work of public health practice. Additionally, comorbid conditions, a significant predictor of disease outcome, are not captured. We found that CCDA data have a broader set of clinical fields and have the advantage of providing valuable comorbidity information. Although only small improvements in completeness were achieved, a high match rate to ELR data makes the CCDA a compelling addition to ELR to improve the analytic power of public health data sets. The CCDA had some fields that remained incomplete, indicating that data capture and sharing at the source remain crucial issues for use of these data.

Initiatives to standardize and automate case report form completion have been developed [28] and piloted [29], which have shown promise at reducing the time to complete reporting. Similar to our results, others have found that health information exchanges show value in prepopulating key elements for reporting through automated matching and searches in the patient record [30]. The use of FHIR [31] may provide an additional path for automated public health case reporting and reducing the administrative burden through API-based connections between public health and EHR systems. An example workflow could be the submission of case data via traditional ELR methods to public health agencies, followed by a “pull” of information from EHR systems by public health via FHIR API calls to complete a record. When combined with an ELR-based trigger for a case (eg, sexually transmitted infection cases), an app that executes FHIR-based queries could complete an electronic case report form in 85% of cases [26]. Additionally, all the key components of FHIR-based workflows for public health reporting are often in place [32]. In the recent past, alignment on the US Core Data for Interoperability (USCDI), with use of FHIR standards, has created a baseline for fields, vocabularies, and content that may enhance existing mandates from meaningful use. Our technical architecture supports the use of mandated as well as available data to create a unified public health data set in the data hub.

A feature of our solution is that it supports the central role of local health departments in data aggregation and reporting. An important component of the public health response in many communities is “home rule” for public health agencies [33] or local jurisdiction and control of policy and approach for local health departments. Home rule laws empower local governments to address public health issues and fill gaps in the patchwork of the national and state-based public health response [33]. In the current pandemic, robust local responses that can enable targeted interventions and planning can allow more sophisticated preparedness planning, pandemic control, and epidemiologic analysis.

For the most efficient data exchange, standards for the structure of data sharing and the semantic representation of information are critical. In this context, the technical and nontechnical handshakes and handoffs related to data are key factors in successful programs. In this setting, technical handshakes are the trust relationships between systems to enable data sharing: the ability to use both authenticated API-based transfers and DIRECT mailbox shares accelerated time to implementation for the project. Technical handoffs were the ability to have seamless data parsing because of robust standards implemented via meaningful use. Given the greater coverage of fields in the COVID-19 PUI form by CCDA files, the ability to leverage the CCDA to increase the completeness of overall COVID-19 PUI reporting is a sign of the value of federal standards for clinical data interchange.

Of more importance were the nontechnical handshakes (ie, relationship building and the development of consensus among institutions to enable sharing of data) and handoffs (ie, the partnerships between public and private entities). A data governance committee was essential to promote trust and enabled the scaling of the program to new data sets and deeper information within sets. At a time of a surge in COVID-19 cases, a private and academic partner (Rush University Medical Center) with the technical capacity was able to rapidly implement a solution. Three implications emerge from the system developed in Chicago. First, relationships and collaborations were critical in the setting of the pandemic to ensure success. Second, the role of public health in driving adoption through the use of mandates was also critical. Finally, the existence of standards and API-based data exchange accelerated adoption in the region.

### Limitations

Our efforts were subject to several limitations. First, the solution that was implemented was used in a single public health jurisdiction and was not deployed to multiple locations. We believe that the use of file types that are widely available through federal mandates (CCDA and ELR data) suggests that our approach could be scalable to multiple health departments, but further investigation is required. An additional limitation was the use of a public health mandate to encourage engagement and participation. Without a requirement for data sharing, lower rates of data sharing likely would have occurred. Finally, although we made significant process in our effort at regional data exchange for public health purposes, much work remains nationally to facilitate scalable data sharing. To avoid the challenges faced in this pandemic with data liquidity, more work is needed for automation of data collection and networks of “on-the-ready” data sharing built outside of pandemics.

### Conclusion

We consider this public health and clinical data hub to be an informative example of how common standards across electronic records can be used to create a more complete surveillance record for public health. This report may be a potential template for future extension of the use of standards to improve public health surveillance. Through merging of data, small improvements in completeness were achieved, particularly for comorbidity and hospitalization information for COVID-19 surveillance. A reduction in the administrative burden in reporting remains a goal but will require more broad changes to the US reporting infrastructure.

## Appendix

Multimedia Appendix 1 List of measures and agencies with mandated reporting in April 2020 for COVID-19 in Chicago, Illinois.

## Abbreviations

API: application programming interface
CCDA: consolidated clinical data architecture
CDC: Centers for Disease Control and Prevention
CDPH: Chicago Department of Public Health
ED: emergency department
EHR: electronic health record
ELR: electronic laboratory reporting
FEMA: Federal Emergency Management Agency
FHIR: Fast Healthcare Interoperability Resources
HAN: Health Alert Network
HL7: Health Level Seven International
ICU: intensive care unit
IDPH: Illinois Department of Public Health
I-NEDSS: Illinois National Electronic Disease Surveillance System
NHSN: National Healthcare Safety Network
PUI: Person Under Investigation
REDCap: Research Electronic Data Capture
XDS: cross-enterprise document sharing
