# Supplementary material for: A Standard-Based Citywide Health Information Exchange for Public Health in Response to COVID-19: Development Study
Source: JMIR Public Health Surveill. 2022 Sep 27;8(9):e35973. doi: 10.2196/35973 (PMC9518711; doi:10.2196/35973)
Supplement: Multimedia Appendix 1 [file publichealth_v8i9e35973_app1.docx]

**Multimedia Appendix 1. List of measures and agencies with mandated reporting in April 2020 for COVID-19 in Chicago, Illinois.**

| ﻿Type | Name | Definition | Agency/system |
| --- | --- | --- | --- |
| Bed usage | EMResource^a^ - Bed Availability: Adult Surge ICU^b^ | Number of adult surge ICU beds available at a facility. | EMResource |
| Bed usage | EMResource - Bed Availability: Burn | Availability of burn beds. These are thought of as burn ICU beds, either approved by the American Burn Association or self-designated. These beds are *not* to be included in other ICU bed counts. | EMResource |
| Bed usage | EMResource - Bed Availability: Med/Surg^c^ | Availability of medical/surgical beds. These are also thought of as ward beds. These beds may or may not include cardiac telemetry capability. | EMResource |
| Bed usage | EMResource - Bed Availability: NegFlow^d^ | Availability of negative airflow isolation beds. These provide respiratory isolation. *Note*: This value may represent available beds included in the counts of other types. | EMResource |
| Bed usage | EMResource - Bed Availability: OR^e^ | Availability of ORs that are equipped, staffed, and could be made available for patient care in a short period. | EMResource |
| Bed usage | EMResource - Bed Availability: Other | Number of other available beds. | EMResource |
| Bed usage | EMResource - Bed Availability: Pediatric Surge ICU | Number of pediatric surge beds available at a facility. | EMResource |
| Bed usage | EMResource - Bed Availability: Peds^f^ | Availability of pediatrics beds. These are ward medical/surgical beds for patients 17 years old or younger. | EMResource |
| Bed usage | EMResource - Bed Availability: Psych^g^ | Availability of psych beds. These are ward beds on a closed/locked psych unit or ward beds where a patient will be attended by a sitter. | EMResource |
| Bed usage | EMResource - Bed Available: Adult ICU | Availability of adult ICU beds. These can support critically ill or injured patients, including ventilator support. | EMResource |
| Bed usage | EMResource - Bed Available: NICU^h^ | Number of available NICU beds. | EMResource |
| Bed usage | EMResource - Bed Available: Pediatric ICU | Availability of pediatric ICU beds, similar to adult ICU beds but for patients 17 years old or younger. | EMResource |
| Bed usage | EMResource - Bed Capacity: Adult ICU | Total number of staffed adult ICU beds. These can support critically ill or injured patients, including ventilator support. | EMResource |
| Bed usage | EMResource - Bed Capacity: Burn | Total number of staffed burn beds. These are thought of as burn ICU beds, either approved by the American Burn Association or self-designated. These beds are *not* to be included in other ICU bed counts. | EMResource |
| Bed usage | EMResource - Bed Capacity: ED^i^ | Number of licensed ED beds. | EMResource |
| Bed usage | EMResource - Bed Capacity: Medical/Surgical | Total number of staffed medical/surgical beds. These are also thought of as ward beds. These beds may or may not include cardiac telemetry capability. | EMResource |
| Bed usage | EMResource - Bed Capacity: NegFlow Isolation | Total number of staffed negative airflow isolation beds. These provide respiratory isolation. *Note*: This value may represent available beds included in the counts of other types. | EMResource |
| Bed usage | EMResource - Bed Capacity: NICU | Number of licensed NICU beds. | EMResource |
| Bed usage | EMResource - Bed Capacity: OR | Total number of staffed ORs that are equipped and could be made available for patient care in a short period. | EMResource |
| Bed usage | EMResource - Bed Capacity: Other | Number of other licensed beds. | EMResource |
| Bed usage | EMResource - Bed Capacity: Pediatric ICU | Total number of staffed pediatric ICU beds, similar to adult ICU beds, but for patients 17 years old or younger. | EMResource |
| Bed usage | EMResource - Bed Capacity: Pediatrics | Total number of staffed pediatrics beds. These are ward medical/surgical beds for patients 17 years old or younger. | EMResource |
| Bed usage | EMResource - Bed Capacity: Psych | Total number of staffed psych beds. These are ward beds on a closed/locked psych unit or ward beds where a patient will be attended by a sitter. | EMResource |
| Bed usage | EMResource - Ventilators Available Surge | Number of available surge ventilators at a facility. | EMResource |
| Bed usage | FEMA^j^ - All Hospital Beds | Total number of all staffed inpatient and outpatient beds in your hospital, including all overflow and surge/expansion beds used for inpatients and for outpatients (includes all ICU beds). | FEMA Daily Report |
| Bed usage | FEMA - Hospital Inpatient Bed Occupancy | Total number of staffed inpatient beds that are occupied. | FEMA Daily Report |
| Bed usage | FEMA - Hospital Inpatient Beds | Total number of staffed inpatient beds in your hospital, including all overflow and surge/expansion beds used for inpatients (includes all ICU beds). | FEMA Daily Report |
| Bed usage | FEMA - Hospital Onset | Patients currently hospitalized in inpatient beds with onset of suspected or confirmed COVID-19 14 or more days after hospital admission due to a condition other than COVID-19. | FEMA Daily Report |
| Bed usage | FEMA - Hospitalized and Ventilated COVID Patient | Patients currently hospitalized in inpatient beds who have suspected or confirmed COVID-19 and are on a mechanical ventilator. | FEMA Daily Report |
| Bed usage | FEMA - Hospitalized COVID Patients | Patients currently hospitalized in inpatient beds who have suspected or confirmed COVID-19. | FEMA Daily Report |
| Bed usage | FEMA - ICU Bed Occupancy | Total number of staffed inpatient ICU beds that are occupied. | FEMA Daily Report |
| Bed usage | FEMA - ICU Beds | Total number of staffed inpatient ICU beds. | FEMA Daily Report |
| Bed usage | FEMA - Total Beds (Temporary) | The current number of physical usable beds in the facility. If a bed is not currently staffed but is usable and has the potential to be staffed, it should be counted. The same would apply to a blocked bed. If a bed is currently blocked but is a usable bed, it should be counted. | FEMA Daily Report |
| Bed usage | FEMA - Total Occupied Beds (Temporary) | The number of beds currently occupied with patients. This also includes any patient that may be located in an outpatient area within the facility (eg, ED or postanesthesia care unit [PACU] bays) that have an inpatient or observation order). | FEMA Daily Report |
| Bed usage | ING^k^ - Airborne Isolation Beds | Total number of airborne infection isolation room (AIIR) beds at the hospital. | Illinois National Guard Survey |
| Bed usage | ING - Airborne Isolation Beds Available | Total number of AIIR (airborne infection isolation room) beds available at the hospital. | Illinois National Guard Survey |
| Bed usage | ING - Alternative Care Site - Isolation | Does this facility have isolation capabilities? | Illinois National Guard Survey |
| Bed usage | ING - Alternative Care Site - Respiratory Illness/Ventilation | Can this facility handle patients with respiratory illness (ventilation)? | Illinois National Guard Survey |
| Bed usage | ING - ICU Beds | Total number of ICU beds at the hospital. | Illinois National Guard Survey |
| Bed usage | ING - ICU Beds Available | Total number of ICU beds available at the hospital. | Illinois National Guard Survey |
| Bed usage | ING - Medical/Surgical Beds | Total number of medical/surgical beds at the hospital. | Illinois National Guard Survey |
| Bed usage | ING - Medical/Surgical Beds Available | Total number of medical/surgical beds available at the hospital. | Illinois National Guard Survey |
| Bed usage | ING - Other Staffed Beds | Total number of other staffed beds at the hospital. | Illinois National Guard Survey |
| Bed usage | ING - Other Staffed Beds Available | Total number of other staffed beds available at the hospital. | Illinois National Guard Survey |
| Bed usage | ING - Unlicensed Beds | Does the hospital have unlicensed beds that could be opened if allowed by IDPH^l^ hospital licensing? | Illinois National Guard Survey |
| Bed usage | ING - Unlicensed Beds - Count | How many beds could be opened? | Illinois National Guard Survey |
| Bed usage | NHSN^m^ - All Hospital Beds | Enter the total number of all hospital beds, including inpatient and outpatient beds. All staffed, licensed, and overflow and surge/expansion beds used for inpatients or outpatients. This includes ICU beds. | NHSN Daily Report |
| Bed usage | NHSN - Deaths | Enter the number of patients with suspected or confirmed COVID-19 who died in the hospital, ED, or any overflow location. This includes patients with laboratory-confirmed or clinically diagnosed COVID-19. Please enter the count of deaths newly occurred, at the time the data are collected instead of the cumulated number of deaths. | NHSN Daily Report |
| Bed usage | NHSN - ED/Overflow | Enter the number of patients with suspected or confirmed COVID-19 who are in the ED or any overflow/expansion location awaiting placement in an inpatient bed at the time the data are collected. This includes patients with laboratory-confirmed or clinically diagnosed COVID-19. Overflow locations include any physical locations created to accommodate patients including but not limited to 24-hour observation units, hallways, parking lots, or tents. | NHSN Daily Report |
| Bed usage | NHSN - ED/Overflow and Ventilated | Enter the number of patients with suspected or confirmed COVID-19 who are in the ED or any overflow/expansion location on a mechanical ventilator^n^ at the time the data are collected. This includes patients with laboratory-confirmed or clinically diagnosed COVID-19. | NHSN Daily Report |
| Bed usage | NHSN - Hospital Inpatient Beds | Required. Enter the total number of all inpatient beds, including all staffed, licensed, and overflow and surge/expansion beds created for inpatient care. This includes ICU beds. | NHSN Daily Report |
| Bed usage | NHSN - Hospital Inpatient Beds Occupancy | Enter the total number of staffed inpatient beds occupied by patients at the time the data are collected, including all staffed, licensed, and overflow and surge/expansion beds created for inpatient care. This includes ICU beds. | NHSN Daily Report |
| Bed usage | NHSN - Hospital Onset | Enter the number of patients hospitalized in inpatient beds at the time the data are collected with onset of suspected or confirmed COVID-19 14 or more days after hospitalization (admission date=hospital day 1). This includes laboratory-confirmed or clinically diagnosed COVID-19 cases. | NHSN Daily Report |
| Bed usage | NHSN - Hospitalized | Enter the number of patients hospitalized in inpatient beds at the time the data are collected who have suspected or confirmed COVID-19. This includes the patients with laboratory-confirmed or clinically diagnosed COVID-19. *Confirmed*: A patient with a laboratory-confirmed COVID-19 diagnosis *Suspected*: A patient without a laboratory-confirmed COVID-19 diagnosis who, in accordance with the CDC’s^o^ Interim Public Health Guidance for Evaluating Persons Under Investigation (PUIs^p^), has signs and symptoms compatible with COVID-19 (most patients with confirmed COVID-19 develop fever or symptoms of acute respiratory illness, such as cough, shortness of breath, or myalgia/fatigue). | NHSN Daily Report |
| Bed usage | NHSN - Hospitalized and Ventilated | Enter the number of patients hospitalized in inpatient beds who have suspected or confirmed COVID-19 and are currently on a mechanical ventilator at the time the data are collected. This includes the patients with laboratory-confirmed or clinically diagnosed COVID-19. | NHSN Daily Report |
| Bed usage | NHSN - ICU Bed Occupancy | Enter the total number of staffed ICU beds occupied by patients at the time the data are collected. | NHSN Daily Report |
| Bed usage | NHSN - ICU Beds | Enter the total number of staffed ICU beds. | NHSN Daily Report |
| ED usage | EMResource - Bed Availability: ED | Please add your ED bed availability. | EMResource |
| ED usage | EMResource - Decon Throughput | The number of patients a facility can decontaminate every hour. | EMResource |
| ED usage | FEMA - ED/Overflow | Patients with suspected or confirmed COVID-19 who currently are in the ED or any overflow location awaiting an inpatient bed. | FEMA Daily Report |
| ED usage | FEMA - ED/Overflow and Vented | Patients with suspected or confirmed COVID-19 who currently are in the ED or any overflow location awaiting an inpatient bed and on a mechanical ventilator. | FEMA Daily Report |
| ED usage | ING - Alternative Care Site - Availability | Has the hospital designated (or have access to a site to serve as) an alternate care site (remote location the hospital can stand up to handle a surge of patients)? | Illinois National Guard Survey |
| ED usage | ING - Alternative Care Site - Operation | Is this facility operational? | Illinois National Guard Survey |
| ED usage | ING - External Triage/Screening - Availability | Does the hospital have an external triage/screening area away from the ED or outside the hospital? | Illinois National Guard Survey |
| ED usage | ING - External Triage/Screening - Operation | Is the external triage/screening area in operation? | Illinois National Guard Survey |
| ED usage | ING - Hospital Entrance for COVID Patients | Has the hospital identified an area for potential COVID-19 patients to enter the hospital, other than the ED? | Illinois National Guard Survey |
| Lab testing | FEMA - COVID-19 Patients Confirmed (Temporary) | The number of patients anywhere in the facility who are currently positive with COVID-19. This does not include patients who were once positive but are now negative. | FEMA Daily Report |
| Lab testing | FEMA - COVID-19 Patients Under Investigation (Temporary) | The number of patients anywhere in the facility who are currently under investigation suspected to have COVID-19. | FEMA Daily Report |
| Lab testing | FEMA - Cumulative Diagnostic Tests Ordered/Received | All tests ordered to date; rush internal tests only. | FEMA Daily Report |
| Lab testing | FEMA - Cumulative Negative COVID-19 Tests | All negative test results released to date; rush internal tests only. | FEMA Daily Report |
| Lab testing | FEMA - Cumulative Positive COVID-19 Tests | All positive test results released to date; rush internal tests only. | FEMA Daily Report |
| Lab testing | FEMA - Cumulative Tests Performed | All tests with results released to date; rush internal tests only. | FEMA Daily Report |
| Lab testing | FEMA - New Diagnostic Tests Ordered/Received | Midnight to midnight cut-off, tests ordered on previous date queried; rush internal tests only. | FEMA Daily Report |
| Lab testing | FEMA - New Negative COVID-19 Tests | Midnight to midnight cut-off, negative test results released on previous date queried; rush internal tests only. | FEMA Daily Report |
| Lab testing | FEMA - New Positive COVID-19 Tests | Midnight to midnight cut-off, positive test results released on previous date queried; rush internal tests only. | FEMA Daily Report |
| Lab testing | FEMA - New Tests Resulted | Midnight to midnight cut-off, test results released on previous date queried; rush internal tests only. | FEMA Daily Report |
| Other | FEMA - Deaths | Number of patients with suspected or confirmed COVID-19 who died in the hospital, ED, or any overflow location on the date for which you are reporting. | FEMA Daily Report |
| Other | ING - Security | Is there a security need? | Illinois National Guard Survey |
| Supplies | FEMA - On-hand Supply of N95 Masks | 0 days _ 1-3 days _ 4-14 days _ 15 or more days. | FEMA Daily Report |
| Supplies | ING - Alternative Care Site - Additional Resources | Are additional resources needed to operate the facility? | Illinois National Guard Survey |
| Supplies | ING - Alternative Care Site - Establishment | Is there a need for the state to assist with the establishment? | Illinois National Guard Survey |
| Supplies | ING - Alternative Care Site - Support | What support does the hospital need to operate the facility? | Illinois National Guard Survey |
| Supplies | ING - Blood Shortage | Does the hospital have blood shortage? | Illinois National Guard Survey |
| Supplies | ING - Critical Resource Needs - Current | What are your critical resource and support needs currently? | Illinois National Guard Survey |
| Supplies | ING - Critical Resource Needs - Next 12 hours | What are your critical resource and support needs in the next 12 hours? | Illinois National Guard Survey |
| Supplies | ING – PPE^q^ - Face Shields Inventory | How many face shields do you have on hand? | Illinois National Guard Survey |
| Supplies | ING - PPE - Face Shields Request | How many face shields do you need to request? | Illinois National Guard Survey |
| Supplies | ING - PPE - Gloves Inventory | How many gloves do you have on hand? | Illinois National Guard Survey |
| Supplies | ING - PPE - Gloves Request | How many gloves do you need to request? | Illinois National Guard Survey |
| Supplies | ING - PPE - Gowns Inventory | How many gowns do you have on hand? | Illinois National Guard Survey |
| Supplies | ING - PPE - Gowns Request | How many gowns do you need to request? | Illinois National Guard Survey |
| Supplies | ING - PPE - Masks Inventory | How many masks do you have on hand? | Illinois National Guard Survey |
| Supplies | ING - PPE - Masks Request | How many masks do you need to request? | Illinois National Guard Survey |
| Supplies | ING - PPE - Thermometers Request | How many thermometers do you need to request? | Illinois National Guard Survey |
| Supplies | ING - PPE - Thermometers Inventory | How many thermometers do you have on hand? | Illinois National Guard Survey |
| Supplies | ING - PPE Request | Did you submit a resource request to the county for PPE for your facility? | Illinois National Guard Survey |
| Supplies | ING - Supplies for EMS^r^ | Is the hospital able to replace supplies for EMS providers sent to the ED and for inpatients? | Illinois National Guard Survey |
| Supplies | ING - Surge Situation | Is the hospital in a surge situation? | Illinois National Guard Survey |
| Supplies | ING - Urgent Resource Requests | Does the hospital have any urgent (ie, needed withing 6 hours) resource requests? | Illinois National Guard Survey |
| Ventilator use | EMResource - Available: Vents^s^ | Total number of full-feature ventilators available to the facility that can support patients >5 kg and above. | EMResource |
| Ventilator use | EMResource - Capacity: Vents | Total number of ventilators at this facility (in use and available). | EMResource |
| Ventilator use | FEMA - COVID-19 Patients Using Ventilation (Temporary) | The current number of confirmed COVID-19 patients (laboratory confirmed or clinically diagnosed) using ventilators. | FEMA Daily Report |
| Ventilator use | FEMA - Mechanical Ventilators | Total number of ventilators available. | FEMA Daily Report |
| Ventilator use | FEMA - Mechanical Ventilators in Use | Total number of ventilators in use. | FEMA Daily Report |
| Ventilator use | ING - Ventilators | Total number of ventilators in use at the hospital. | Illinois National Guard Survey |
| Ventilator use | ING - Ventilators Available | Total number of ventilators available at the hospital. | Illinois National Guard Survey |
| Ventilator use | NHSN - Mechanical Ventilators in Use | Enter the total number of mechanical ventilators in use at the time the data are collected, including anesthesia machines and portable/transport ventilators. Include BiPAP machines if the hospital uses BiPAP to deliver positive pressure ventilation via artificial airways. | NHSN Daily Report |
| Ventilator use | NHSN - Mechanical Ventilators | Enter the total number of mechanical ventilators, including anesthesia machines and portable/transport ventilators, available in the facility. Include BiPAP machines if the hospital uses BiPAP to deliver positive pressure ventilation via artificial airways. | NHSN Daily Report |

^a^EMResource: software tool used for reporting bed capacity to health departments.

^b^ICU: intensive care unit.

^c^Med/Surg: medical or surgical unit.

^d^NegFlow: negative airflow room.

^e^OR: operating room.

^f^Peds: pediatrics.

^g^Psych: psychiatry.

^h^NICU: neonatal intensive care unit.

^i^ED: emergency department.

^j^FEMA: Federal Emergency Management Agency.

^k^ING: Illinois National Guard.

^l^IDPH: Illinois Department of Public Health.

^m^NHSN: National Healthcare Safety Network.

^n^Ventilator: Any device used to support, assist, or control respiration (inclusive of the weaning period) through the application of positive pressure to the airway when delivered via an artificial airway, specifically an oral/nasal endotracheal or tracheostomy tube. *Note*: Ventilation and lung expansion devices that deliver positive pressure to the airway (eg, continuous positive airway pressure [CPAP], bilevel positive airway pressure [BiPAP], bilevel, intermittent positive pressure breathing [IPPB], and positive end-expiratory pressure [PEEP]) via noninvasive means (eg, nasal prongs, nasal mask, full face mask, total mask) are not considered ventilators unless positive pressure is delivered via an artificial airway (oral/nasal endotracheal or tracheostomy tube).

^o^CDC: Centers for Disease Control and Prevention.

^p^PUI: Person Under Investigation.

^q^PPE: personal protective equipment.

^r^EMS: emergency medical services.

^s^Vents: ventilators.
